# Effect of Structured Phenolic Lipids with EPA/DHA and Gallic Acid against Metabolic-Associated Fatty Liver Disease (MAFLD) in Mice

**DOI:** 10.3390/molecules27227702

**Published:** 2022-11-09

**Authors:** Gretel Dovale-Rosabal, Alejandra Espinosa, Alicia Rodríguez, Andrés Barriga, Alan Palomino-Calderón, Nalda Romero, Rodrigo Hernán Troncoso, Santiago Pedro Aubourg

**Affiliations:** 1 Department of Food Science and Chemical Technology, Faculty of Chemical and Pharmaceutical Sciences, University of Chile, Carlos Lorca Tobar 964, Santiago 8380494, Chile; 2 Escuela de Medicina, Campus San Felipe, Universidad de Valparaíso, Valparaíso 2340000, Chile; 3 Department of Medical Technology, Faculty of Medicine, University of Chile, Independencia 1027, Santiago 8380000, Chile; 4 Centre of Studies for the Development of Chemistry (CEPEDEQ), Faculty of Chemical and Pharmaceutical Sciences, University of Chile, Carlos Lorca Tobar 964, Santiago 8380494, Chile; 5 Laboratory of Nutrition and Physical Activity (LABINAF), Institute of Nutrition and Food Technology (INTA), Universidad de Chile, El Líbano 5524, Santiago 7830490, Chile; 6 Department of Food Technology, Marine Research Institute (CSIC), Eduardo Cabello 6, 36208 Vigo, Spain

**Keywords:** obesity, metabolic (dysfunction)-associated fatty liver disease (MAFLD), obese mice, high-fat diet (HFD), structured acylglycerols (sAG), gallic acid (GA), EPA and DHA, enzymatic acidolysis, CO_2_ supercritical conditions, structured phenolic acylglycerols (sPAG)

## Abstract

Obesity is the leading risk factor for developing metabolic (dysfunction)-associated fatty liver disease (MAFLD). The food industry has an essential role in searching for new strategies to improve primary food sources to revert some of the metabolic alterations induced by obesity. There is consistent evidence that long-chain polyunsaturated fatty acids (n-3 LCPUFA) belonging to the n-3 series, i.e., eicosapentaenoic (20:5n-3, EPA) and docosahexaenoic (22:6n-3, DHA) acids, could revert some alterations associated with obesity-induced metabolic diseases. A relevant tool is the synthesis of structured acylglycerols (sAG), which include EPA or DHA at the sn-2 position. On the other hand, it has been reported that a crucial role of antioxidants is the reversion of MAFLD. In this work, we studied the effects of new molecules incorporating gallic acid (GA) into EPA/DHA-rich structured lipids. Mice were fed with a high-fat diet (60%) for three months and were then divided into five groups for supplementation with sAG and sAG structured with gallic acid (structured phenolic acylglycerols, sPAG). sPAG synthesis was optimized using a 2²-screening factorial design based on the response surface methodology (RSM). Our results show that treatment of sPAG was effective in decreasing visceral fat, fasting glycemia, fasting insulin, suggesting that this new molecule has a potential use in the reversal of MAFLD-associated alterations.

## 1. Introduction

Obesity is the leading risk factor for developing metabolic (dysfunction)-associated fatty liver disease (MAFLD). Metabolism-associated fatty liver disease (MAFLD) is currently the most common cause of liver disease worldwide. MAFLD is characterized by hepatic steatosis under a background of overweight or obesity and insulin resistance [1,2,3]. This entity was previously known as non-alcoholic fatty liver disease (NAFLD), but today it is impossible to isolate the alcoholic component in its etiology [4]. In the proposed new definition for the diagnosis of MAFLD, the criteria are based on evidence of hepatic steatosis, in addition to one of the following three criteria, namely overweight/obesity, presence of type 2 diabetes mellitus, or evidence of metabolic dysregulation [1]. The following steps of this disease is incipient steatohepatitis and then cirrhosis. The pathophysiology involved in this chain of events includes the lipoperoxidation of intracellular lipid droplets, oxidative stress, inflammation, and the activation of the fibrosis gene expression program [5]. The severity of this poor prognosis challenges us to find strategies against disease progression. Several alimentary supplementations are currently being investigated for improving obesity-triggered metabolic alterations.

One of the most promising strategies has been the incorporation of n-3 long-chain polyunsaturated fatty acids (n-3 LCPUFA) into the diet with a lifestyle change, such as weight loss [6,7]. Additionally, some cohort studies about EPA and DHA effects have demonstrated physiological benefits on blood pressure, heart rate, and inflammation, among others. Furthermore, in animal models, a significant body of evidence points to the role of EPA and DHA incorporation as metabolic mediators, decreasing the steatosis status in hepatocytes [6,8]. n-3 LCPUFA, such as EPA and DHA, produce essential health benefits, manifesting their role as bioactive lipid mediators of the protective action exerted by diets rich in these compounds [9]. Abundant benefits have been attributed to dietary n-3 LCPUFA, including protection against cardiac arrhythmia, triglyceride-lowering, and ameliorating inflammatory and neurodegenerative disorders. Marine oils are widely used due to their high content of EPA and DHA, and their consumption is associated with a low prevalence of cardiac, circulatory and inflammatory diseases [10]. A low n-6/n-3 ratio, together with daily consumption of n-3 LCPUFA, is related to a lower prevalence of chronic non-communicable diseases (NCDs) [11] and decreased mortality and cardiac risk [12].

EPA and DHA are substrates for the formation of a series of lipid derivatives, such as prostaglandins of series 3 and leukotrienes of series 5 called eicosanoids (derived from EPA) and docosanoids (DHA derivatives), which exert important functions in cellular metabolism [13,14]. Considering that obesity produces an intracellular oxidant environment, increasing the oxidized glutathione ratio (GSH/GSSG), affecting lipid molecules stored as lipid droplets, antioxidants incorporation in the diet could prevent or revert the damage induced by lipoperoxidation in the liver. Antioxidant molecules can revert lipid peroxidation reactions, inhibit lipoxygenase and scavenge free radicals inside the cell. In this context, gallic acid (GA; 3,4,5-trihydroxybenzoic acid) and its derivatives of hydroxybenzoic acids are natural polyphenol products with high antioxidant capacity, and recently there has been an increased interest in gallic acid as a promissory therapy against MAFLD [15,16,17]. Current work aims to test the effect of phenolic lipids structured with gallic acids in the reversion of metabolic alteration HFD-induced in mice. Antioxidants used in foodstuffs to protect fats against oxidative rancidity are phenolic compounds: acid-phenols or flavonoids and their esters. GA and its derivatives are natural polyphenol products, which are found to be particularly abundant in processed beverages, such as red wines and green teas [18,19], including walnuts, raspberry, black currant, and strawberry. They are natural antioxidants in plant cell walls [20] with anti-inflammatory activities [21]. They have radical scavenging, anti-oxidative, anti-fungal [22], anti-cancer [23], and chemoprotective [24] properties, which are of great importance to the food, pharmaceutical, and drug industries [25,26]. The bioactivity of phenolic molecules may be related to their antioxidant behavior, attributed to their ability to chelate metals, inhibit lipoxygenase and scavenge free radicals. These findings may provide a pharmacological basis for GA to be used as an antioxidant metabolite for the treatment of neurodegenerative diseases, such as hippocampal degeneration [27], hyperglycemia [28], and NAFLD [29]. Incorporating phenolic acids as GA in triacylglycerol (TG) could give rise to structured phenolic acylglycerols. These new molecules could benefit from the combined properties of phenols and n-3 LCPUFAs. Nevertheless, the use of phenolic acids as antioxidants in fat and oil systems is limited by their hydrophilic nature [30,31]; then, improvements or changes in the solubility and miscibility characteristics of phenolic compounds can be achieved after their incorporation into structured TG [32]. Phenolic lipids have been synthesized through transesterification to improve lipid solubility [33,34]. Structured acylglycerols (sAG) or structured lipids (SL), whose composition and positional distribution of fatty acids (FA) in the glycerol skeleton have been modified, can be obtained by enzymatic catalysis reactions and genetic engineering. As a result, “tailor-made” lipids can be obtained with favorable physical characteristics, chemical properties, and nutritional benefits. This structuring can be done through chemical or enzymatic transesterification reactions by altering the composition and distribution of FA in the glycerol molecule [14,35,36,37,38,39,40]. Lipases can be used in supercritical media (SC) to produce sAG since the increase in the solubility of lipid and hydrophobic substrates in non-polar media produces reverse reactions to hydrolysis, favoring enzymatic synthesis such as esterification, interesterification, alcoholysis and acidolysis [41,42]. Among SC fluids, carbon dioxide (CO_2_) is an ideal solvent for food application enzymatic synthesis. It is considered a GRAS compound [43], has a variable density and great solvent power, and can be easily separated from the reaction medium by depressurization. Sabally et al. [44] reported the enzymatic synthesis of phenolic lipids by lipase-catalyzed transesterification of dihydrocaffeic acid (DHCA) with flaxseed oil in a selected organic solvent medium. These synthesized phenolic lipids have potential use as nutraceutical products. However, there are no reports concerning the production of structured phenolic acylglycerols (sPAG) that include gallic acid and EPA or DHA under CO_2_SC conditions suitable for reversion of metabolic abnormalities present in obesity of mice. The present investigation aimed to study the effect of EPA/DHA and gallic acid structured phenolic acylglycerols by enzymatic acidolysis in CO_2_SC media to enhance against metabolic-associated fatty liver disease (MAFLD) in mice. Our finding supports that the four-week treatment with structured phenolic lipids could revert liver steatosis and insulin resistance in obese mice. 

## 2. Results

### 2.1. Acidolysis Process for Obtaining sPAG from sAG by 2²-Screening Factorial Design

The sAG was subjected to an experimental process of enzymatic acidolysis to obtain sPAG and optimize its content of EPA, DHA, and the incorporation of gallic acid in the lipid. Table 1 shows the conditions of the 2²-screening factorial design based on the response surface methodology (RSM), whose independent variables were: SC pressure and SC temperature. The response variables where: EPA, DHA, EPA+DHA content, and sPAG enzymatic synthesis. Based on the RSM, a 2²-screening factorial design of two factors (SC pressure and SC temperature) and seven runs with three replicates of the central point were used to estimate the experimental error (Table 1). The range of the design variables was 40–80 °C (SC temperature) and 78–300 bar (SC pressure).

Equations (1)–(4) present the proposed models of the response variables expressed in (g/100 g TFA) EPA content, DHA content, EPA + DHA content, and sPAG synthesis (%) for the levels of two factors (SC temperature and SC pressure) using a first-degree polynomial. Models of sPAG synthesis in terms of their regression coefficients for the dependent variables were the following:

EPA = 10.6364 + 0.0024423∗A − 0.00323679∗B + 0.0000792786∗AB(1)

DHA = 6.46687 + 0.0239618∗A + 0.0020332∗B − 0.0000132802∗AB(2)

EPA + DHA = 17.1033 + 0.0264041∗A − 0.00120359∗B + 0.0000659985∗AB(3)

sPAG Synthesis = 23.2447 + 0.153716∗A − 0.018018∗B − 0.000304054∗AB(4)

The four models had a coefficient of determination (R² adjusted for degrees of freedom) of 98.64%; 84.31%; 96.91%, and 77.16% for EPA, DHA, EPA + DHA, and sPAG synthesis, respectively; this indicated that they adequately represent the variability of the data.

**Figure 1:**
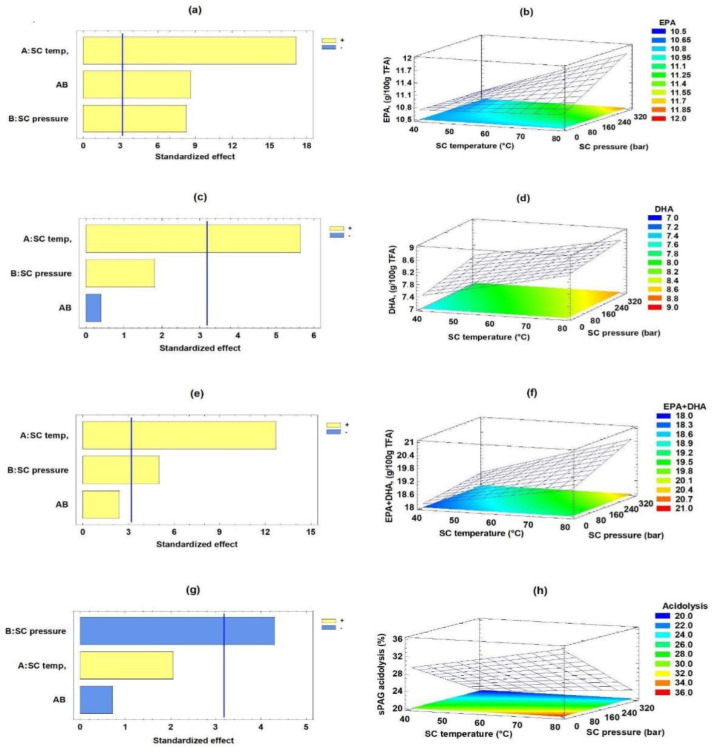
Effect of CO_2_SC temperature and CO_2_SC pressure on the sPAG synthesis process. Standardized Pareto diagrams. Panels (**a**,**c**,**e**,**g**), (EPA, DHA and EPA + DHA content (g/100 g TFA) and phenolic acidolysis (%), respectively). Estimated response surface and contour response surface. Panels (**b**,**d**,**f**,**h**), (EPA, DHA and EPA + DHA content (g/100 g TFA), phenolic acidolysis (%) content, respectively).

Figure 1 shows the main independent variables that affect the enzymatic acidolysis process using Pareto-type graphs, response surface, and contour response surface. The independent variables that significantly influenced the content of EPA, DHA and EPA + DHA on sPAG synthesis were determined by statistical analysis using RSM. Pareto-type graphs show the linear effect and interaction for each response variable in decreasing order (the line marks *p* ˂ 0.05). The standardized Pareto diagram (Figure 1a) shows that both temperature and SC pressure, as well as their interaction, had significant effects (*p* < 0.05) on EPA content. Figure 1b shows the response surface and contour for incorporating EPA into sPAG as a function of SC pressure and SC temperature. EPA content increased with the increase in SC temperature and SC pressure achieving a maximum at high values of both variables (*p* < 0.05). Figure 1c shows the standardized Pareto diagram for DHA content. Only the SC temperature significantly affected DHA content (*p* < 0.05). Figure 1d shows the response surface and contour for incorporation of DHA, presenting a maximum at high SC temperature (*p* < 0.05) and SC pressure. Figure 1e shows that temperature and SC pressure had significant effects (*p* < 0.05) on EPA + DHA content. It can be observed that both independent variables led to a maximum content of EPA + DHA at high levels (*p* < 0.05) (Figure 1f).

Figure 1g shows that the SC pressure significantly affected the phenolic acidolysis (*p* < 0.05). Figure 1h shows the response surface and contour for phenolic acidolysis as a function of the temperature and SC pressure when the SC pressure variable led to a maximum content of phenolic acidolysis at low levels (*p* < 0.05), while the SC temperature was not significant (*p* > 0.05). Thus, SC pressure was the most crucial factor in phenolic acidolysis, and a low SC pressure leads to an increase in it (*p* < 0.05) (Figure 1g,h). Pando et al. [36] reported the acylglycerol synthesis including EPA and DHA (sTAG) from rainbow trout (*Oncorhynchus mykiss*) belly flap oil and caprylic acid (CA) catalyzed by *Thermomyces lanuginosus* lipase under CO_2_SC. In this case, the synthesis of sTAG with EPA, DHA, and CA under CO_2_SC was significantly affected by the n-3 LCPUFA:CA content ratio and SC time.

### 2.2. Optimization of the Acidolysis Process for Obtaining sPAG by RSM

**Figure 2:**
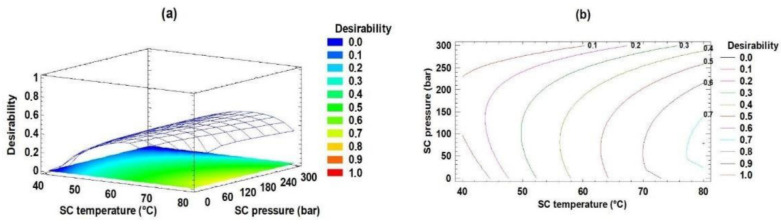
Effect of CO_2_SC temperature and CO_2_SC pressure on the sPAG synthesis. Panels (**a**) desirability function; (**b**) contours of estimated response surface.

The objective of optimizing the independent process variables (pressure and temperature) was to maximize the content of EPA, DHA, and EPA + DHA (g/100 g TFA) in the sPAG and maximize phenolic acidolysis (%). The optimal conditions for EPA, DHA, and EPA + DHA content were 80.0 °C (SC temperature) and 300 bar (SC pressure). The optimal conditions for phenolic acidolysis synthesis were 80.0 °C (SC temperature) and 78 bar (SC pressure). The optimal predicted value for EPA, DHA, EPA + DHA content and sPAG synthesis was 11.76, 8.67, 20.44, and 32.24, respectively. In addition, the joint optimization variables for sPAG synthesis were 80.0 °C and 78 bar, with a maximum desirability of 0.73. As a result, the optimum value of multiple response optimization of the response variables EPA, DHA, EPA + DHA and sPAG synthesis for the enzymatic acidolysis was 11.5, 8.38, 19.53 (g/100 g FA) and 32.24%, respectively(see Table S1 in the Supplementary Materials). Figure 2 shows the behavior of the maximum desirability mentioned, where its increase can be observed as the pressure decreases and the temperature increases.

Pando et al. [36] reported the esterification of EPA or DHA and caprylic acid (C8:0) in structured triacylglycerols (sTAG) using *Thermomyces lanuginosus* lipase as a biocatalyst. In this case, the optimal conditions were established as: 75.5 (n-3 LCPUFA:CA content ratio), 40.0 °C (SC temperature), 300 bar (SC pressure), 3.1 h (time), and 24.0 wt. % (glycerol content). As result, the optimum value of multiple response optimization of the response variables for the synthesis of sTAG was 31.5 (EPA), 19.6 (DHA), and 49.6 of CA (g/100 g TFA).

### 2.3. Validation of the Acidolysis Process for Obtaining sPAG by RSM

Primarily the synthesis of sAG was obtained by enzymatic acidolysis process under CO_2_SC from deodorized refined commercial salmon oil (DRCSO) and n-3 polyunsaturated fatty acids concentrated by inclusion with urea (n-3 PUFAC). The fatty acid composition and quantification of sAG are shown in Table 2 and provide the following decreasing sequence for the most abundant fatty acids (expressed in g/100 g of total fatty acids (TFA)): oleic acid (34.66 ± 0.03), linoleic acid (15.48 ± 0.00), palmitic acid (10.60 ± 0.00), DHA (7.18 ± 0.02), EPA (5.92 ± 0.01) and finally, α-linolenic acid (4.78 ± 0.00).

For the validation of the sPAG synthesis process, the theoretical conditions (78 bar; 80 °C) were taken into account, which predict the maximum value of EPA + DHA and allowed obtaining an optimal sPAG with 19.55 g/100 g TFA (Table 2), increasing its content concerning sAG (namely, 1.49 times).

Table 2 also shows that EPA and DHA individually increased their content by 1.87 and 1.17 times, respectively, while saturated and monounsaturated fatty acids decreased their presence (1.3 and 1.2 times, respectively). The principal fatty acids of the sPAG showed changes versus the sAG with the decrease in the content (expressed in g/100 g TFA) of oleic acid (34.66 vs. 27.60) and palmitic acid (10.6 vs. 7.90) and with the increase in linoleic acid (15.48 vs. 18.12), alpha-linolenic acid (4.78 vs. 5.95), EPA (5.92 vs. 11.11) and DHA (7.18 vs. 8.44). Therefore, the enzymatic acidolysis process in CO_2_SC, optimized by RSM, exchanged the fatty acids of sAG, favoring the incorporation of EPA and DHA into sPAG glycerol.

### 2.4. Structural Analysis of Optimal Validated sPAG by HPLC-UV

**Figure 3:**
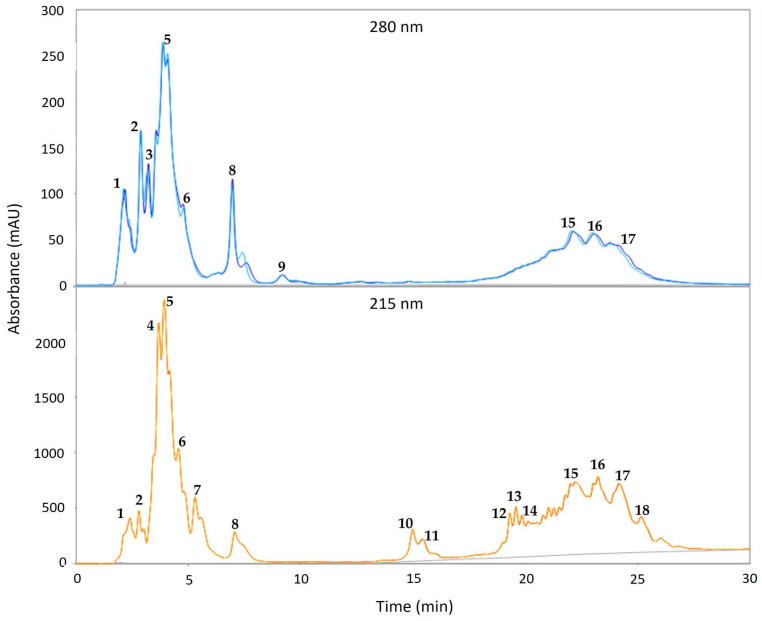
HPLC chromatograms at 280 and 215 nm of the validated optimal sPAG. Peak numbers were identified as follows: gallic acid #1; sAG triacylglycerols #18, phenolic monoacylglycerols #2, #5–6 and #8, phenolic diacylglycerols #15–17, monoacylglycerols #4 and 7, diacylglycerols #10–14, side reaction product #3 and 9.

Validated optimal sPAG was analyzed by HPLC. Figure 3 shows the corresponding elution profile, monitored at 280 nm and 215 nm. Peak #1, which absorbed at both wavelengths (215 and 280 nm), was identified as gallic acid in reference to the standard, while peak #18, absorbing only at 215 nm, corresponds to the TG of the initial sAG. Peaks #2, 5–6 and #8 were characterized as phenolic MG and peaks #15–17 as phenolic DG; they absorbed at both 215 and 280 nm [44]. The hydrolytic by-products of sAG (absorbing only at 215 nm) were represented as peaks #4 and 7 (MG) and peaks #10–14 (DG). Peaks #3 and 9 could be a side reaction product [45,46,47]. These results are in correspondence with what was observed in TLC regarding the synthesis of phenolic acylglycerides (sPAG).

### 2.5. Glucose Homeostasis after the Treatment with sPAG

**Figure 4:**
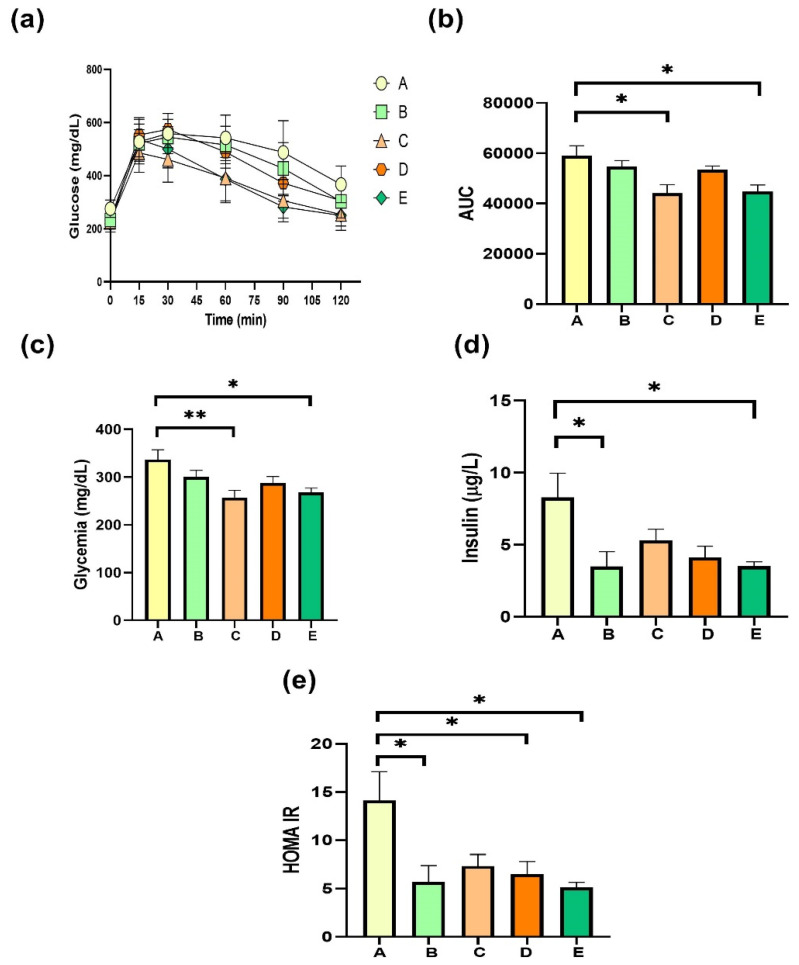
Effects of sPAG treatment on glycemic management in obese mice. (**a**) intraperitoneal glucose tolerance test shows; (**b**) area under curve from (**a**); (**c**) fasting glycemia; (**d**) fasting insulin, and (**e**) homeostatic model assessment for insulin resistance. Treatments were as follows: Group A, sunflower oil; group B, gallic acid; group C, DRCSO; group D, sAG and group E, sPAG. Mice were fasted for 6 h before euthanasia (n = 6–8). Data are expressed as mean ± standard error of the mean (S.E.M.). Statistical differences were determined using one-way ANOVA, followed by Tukey’s post hoc test: * *p* < 0.05, ** *p* < 0.01.

Glucose management is one of the main metabolic alterations in MALFD induced by a high-fat diet; therefore, Figure 4 shows the results obtained concerning glucose homeostasis. After three months of HFD feeding, mice were treated with supplements for four weeks. Results show that the glucose sensibility of mice improved significantly after treatment with DRCSO and sPAG in respect to obese mice (Figure 4a,b). Similarly, fasting glycemia was lower in HFD-fed mice with the same treatments (Figure 4c). Fasting insulin was decreased when obese mice were treated with gallic acid or the sPAG (Figure 4d). HOMA-IR, a mathematical estimation of insulin resistance, was better than group A when the animals were supplemented with gallic acid, sAG, and the sPAG (Figure 4e). These results suggest that four weeks of treatment with lipid-rich structures in n3-PUFA plus gallic acid can potentially improve glucose homeostasis markers in MAFLD.

### 2.6. Gallic Acid Supplementation Decrease MAFLD-Associated Liver Damage

**Figure 5:**
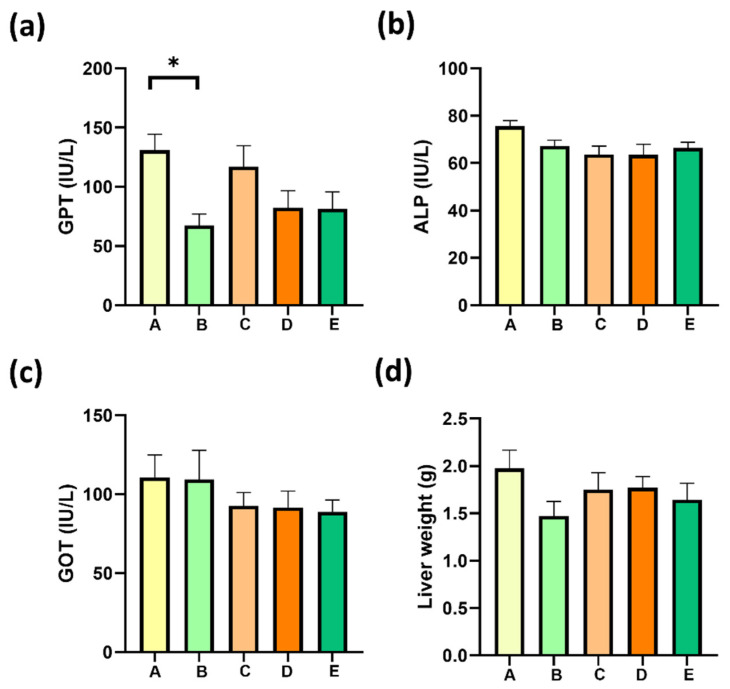
Effects of sPAG treatment on liver markers in obese mice. (**a**) glutamate–pyruvate transaminase; (**b**) alkaline phosphatase; (**c**) glutamic oxaloacetic transaminase (**d**) liver weight. Treatments were as follows: Group A, sunflower oil; group B, gallic acid; C, DRCSO; D, sAG and E, sPAG. Mice were fasted for 6 h before euthanasia (n = 6–8). Data are expressed as mean ± standard error of the mean (S.E.M.). Statistical differences were determined using one-way ANOVA, followed by Tukey’s post hoc test: * *p* < 0.05.

**Figure 6:**
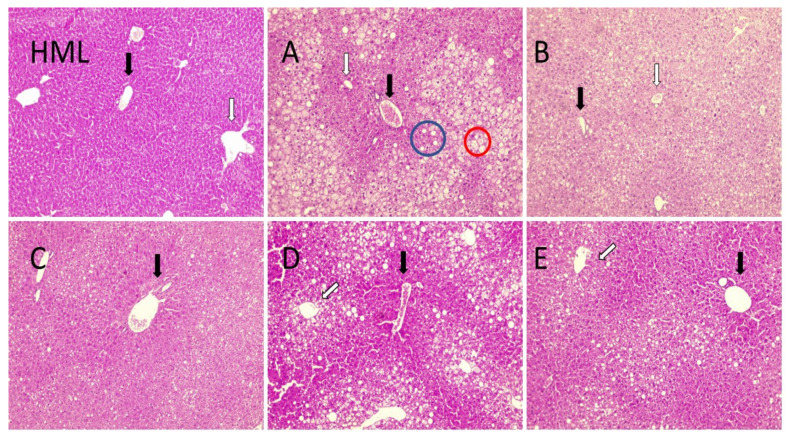
Liver histology of mice treated with a high-fat diet (HFD) supplemented with different treatments. Representative figures were stained with hematoxylin and eosin. The original magnification was 100×. Black arrows indicate the portal area; white arrows show a portal vein; a blue circle surrounds a macrovesicular steatosis area; a red circle includes microvesicular steatosis with fat droplets inside hepatocytes. Healthy mouse liver (HML); following images are representative of each group: Group (**A**), sunflower oil; group (**B**), gallic acid; group (**C**), DRCSO; group (**D**), sAG and group (**E**), sPAG; n = 4 for each group.

The following measurements were made to demonstrate that treatment of obese–-MALFD mice do not worsen the liver damage associated with an HFD. Liver (glutamic oxaloacetic transaminase, GOT, and glutamate–pyruvate transaminase, GPT) or bile ductal (ALP) damage serum markers were measured after the treatment of mice with structured lipids. Neither glutamic oxaloacetic transaminase, alkaline phosphatase, nor glutamate–pyruvate transaminase increased after treatments (Figure 5a–c). Liver weight also was measured, and no changes were detected (Figure 5d). Interestingly, gallic acid treatment induced a significant decrease in serum GPT, showing a potential improvement in liver damage. Following this last result, group B reversed liver damage, showing a lower degree of macrovesicular and microvesicular steatosis. Similarly, group E evidenced significant tissue regeneration around the portal zone (Figure 6, group E, black arrow). Histological analysis showed evident lipid infiltration characterized by macro- and micro-steatosis, with big and isolated lipid droplets or intracellular lipid droplets, respectively, and hepatocyte ballooning. Both phenomena were present in group A compared with a reference image from healthy mice (Figure 6, Group HML).

The morphological study shows that livers from groups B, C, and E underwent little to no steatosis. Group D was the least improved concerning steatosis; however, regeneration nodules were evident, most of them surrounding the portal space (Figure 6, group D, black arrow).

### 2.7. Four Weeks of Treatment with Structured Lipids Supplementation do Not Decrease MAFLD-Associated Fatty Tissue Weight nor Dyslipidemia 

**Figure 7:**
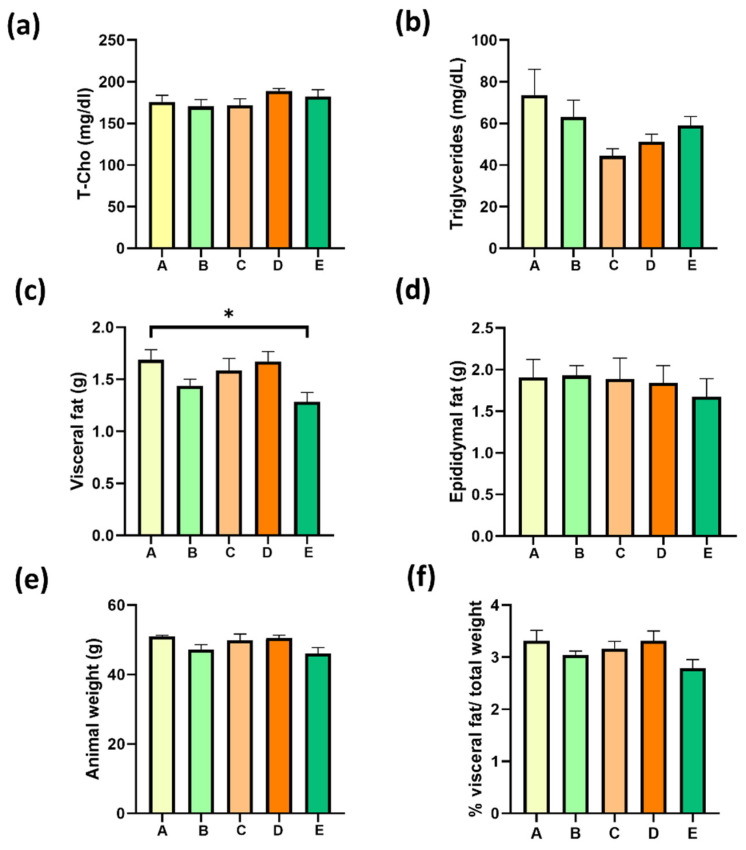
Serum lipid profile and adipose tissue weight of mice treated with a high-fat diet (HFD)-supplemented treatment. (**a**) total cholesterol; (**b**) triglycerides; (**c**) visceral fat; (**d**) epididymal fat; (**e**) animal weight; (**f**) % visceral fat/total weight. Treatments were as follows: Group A, sunflower oil; group B, gallic acid; group C, DRCSO; group D, sAG and group E, sPAG. Mice were fasted for 6 h before euthanasia (n = 6–8). Data are expressed as mean ± standard error of the mean (S.E.M.). Statistical differences were determined using one-way ANOVA, followed by Tukey’s post hoc test: * *p* < 0.05.

MAFLD is characterized by dyslipidemia with a high concentration of triglyceride-rich particles (very-low-density lipoproteins) in serum and a high concentration of low-density lipoproteins (LDL) and low concentration of high-density lipoproteins (HDL); the latter two are parts of total cholesterol levels in serum. Total cholesterol was 176 ± 8 mg/dL in obese mice, and neither supplementation reduced these serum levels (Figure 7a). Total triglycerides were 73 ± 12 mg/dL in the group A; however, no treatment could reduce these levels (Figure 7b).

### 2.8. Four Weeks of Treatment with sPAG Supplementation Decrease Visceral Fat in Obese Mice

Adipose tissue weights were measured to demonstrate the obesity status induced by HFD feeding. Visceral fat was 1.69 g in HFD-fed mice with sunflower oil, corresponding to 48% of the total animal weight (Figure 7e). The visceral fat mass has been associated with insulin resistance in MAFLD. The treatment with sPAG (group E) reduced the visceral fat to 24%, and this was the only group that showed a significant reduction in visceral fat contrasting with the A group (Figure 7c). No significant changes in epididymal fat were observed with any treatment (Figure 7d). Neither total weight nor total fat weight were substantially (*p* > 0.05) changed with any treatment. A slight decrease in the total percentage of fat was obtained within four weeks of sPAG administration (Figure 7f).

## 3. Discussion

In the current times in which obesity has become an epidemic, it is crucial to seek strategies that complement lifestyle changes, thus helping to reverse some of the consequences associated with the metabolic changes generated by obesity. In the present work, we synthesized bioactive lipids that combine the therapeutic properties of n3-LCPUFA and the antioxidant properties of gallic acid in the same molecule. The study performed on mice fed an HFD to promote the onset of insulin resistance, low-grade steatosis, and obesity showed that the model succeeded in representing the conditions present in MAFLD [48,49]. The results showed that the four-week administration of DRCSO alone could improve insulin levels and the HOMA index with respect to a HFD (Figure 4d,e). These results are encouraging, considering that insulin resistance is the hallmark of MAFLD. Furthermore, in our hands, we observed a decrease in fasting insulin with gallic acid treatment (Figure 4d). In another animal model, a promissory effect of gallic acid on glucose management was reported; administration of gallic acid for four weeks also improved insulin resistance index, antioxidant enzymes activities and insulin signaling in high-fructose-diet-fed animal, endorsing its free-radical scavenging properties [50].

It is also promising to note that the structured lipid rich in EPA/DHA and gallic acid (sPAG) achieved results similar to those obtained with DRCSO (Figure 4d). We can conclude that the EPA and DHA enrichment of the diet probably leads to a decrease in fasting insulin levels without the addition of an antioxidant to potentiate this effect. The results obtained with DRCSO agree with the results previously shown in our research group [51,52,53].

Another significant result is that we demonstrated that none of the treatments based on structured lipids were hepatotoxic (Figure 5 and Figure 6). Hepatic steatosis is a characteristic alteration in MALDF, and we could replicate the damage by fatty infiltration of hepatocytes in our animal model. Figure 6a shows an increase in fatty infiltration shown as macro- and micro-steatosis, as has been previously reported [54]. On analyzing the histologic images of the livers of these animals and the levels of transaminases, it was observed that the best effect was obtained in the group treated with gallic acid. These results are in agreement with other authors [55]. Neves Sousa et al. [50] reported that oral administration of gallic acid to rats decreases the gene expression of enzymes related to the induction of hepatic steatosis, such as acetyl-CoA carboxylase (ACC), sterol regulatory element binding protein-1 (SREBP-1) and fatty acid synthase (FAS), an increase associated with HFD feeding.

The importance of our work lies on the fact that it is possible to synthesize lipid molecules rich in DHA/EPA and to include a molecule of gallic acid in its acylglycerol structure. In this strategy, we obtain, in the same compound, a potent antioxidant that, on the one hand, protects the new lipid from oxidation and, on the other hand, provides its own antioxidant capacity to improve the organism’s health. The novelty of this work is the use of SC fluid for the first time to synthetize a new structured phenolic lipid rich in gallic acid, with proven biological activity. Our first approach in a biological model was to evaluate the improvements in the alterations generated by obesity, specifically at the level of MAFLD development. Despite having had positive but discrete results, we consider that these new structured lipids can generate an exciting alternative to enhance their use in reversing some metabolic diseases. Further research would be addressed to apply and evaluate safe and effective doses in animals in more detail, safeguarding the health of the main vital organs; later on, the research ought to be focused on safety in humans.

## 4. Materials and Methods

The study was carried out from structured acylglycerides (sAG), obtained by optimizing the enzymatic acidolysis process using *Candida antarctica* in a CO_2_SC from n-3 polyunsaturated fatty acids concentrated (n-3 PUFAC) and deodorized refined commercial salmon oil (DRCSO). For the enzymatic acidolysis, the following conditions were used: 10 g of a substrate (n-3 PUFAC/DRCSO ratio); SC temperature from 40 to 60 °C; time of 6 hours, and SC pressure from 78 to 300 bar. The amount of lipase Novozyme 435 varied from 0 to 10% of the substrate [56,57]. The n-3 PUFAC concentrate was obtained using DRCSO as the raw material by optimizing the complexation process with urea [51]. DRCSO was obtained from the company Fiordo Austral S.A. (Puerto Montt, Chile) and sunflower oil (*Helianthus annuus*), obtained from the company Natura (Santiago, Chile) as a control, both stored at −80 °C in amber plastic bottles in 250 mL format to facilitate their use. Standards for thin-layer chromatography (TLC) were purchased from Sigma Aldrich, Merck, St. Louis, MO, USA. Lipase (Lipozyme TLL) from *Thermomyces lanuginosus* was donated by Blumos S.A. (Santiago, Chile). Methyl tricosanoate (23:0) internal standard and GLC-463 reference standard for gas chromatography were obtained from Nu-Check-Prep, Elysian, Seattle, WA, USA. CO_2_, N_2_, and H_2_ gas was purchased from Gaslab-Linde (Santiago, Chile). Biological tests: The insulin (mouse) ultrasensitive ELISA test was obtained from the company Mercodia (Uppsala, Sweden). For the determinations of total cholesterol, plasmatic triacylglycerides, glutamic oxalacetic transaminase (GOT), and glutamic pyruvic transaminase (GPT), the in vitro SPOTCHEM II and KENSHIN-2 reactive strips obtained from the company ARKRAY INC., Kyoto, Japan, were used.

### 4.1. Fatty Acid Composition and Quantification by GLC

The fatty acids (FA) profile and EPA/DHA quantification of sAG and sPAG were assessed in a gas–liquid chromatograph (7890B, Agilent, Santiago, Chile) with a fused-silica capillary column (HP-88, 100 m × 0.25 mm i.d. × 0.20 μm film thickness) and a flame ionization detector. A methylation process was performed to obtain FAMEs. The reference standard NU-CHEK GLC463 was used to identify the FA profiles by comparing the retention times [58]. The concentration of the different FAME was determined from the calibration curves by assessment of the peak/area ratio. Finally, the individual FA (g/100 g TFA) was quantified by employing C23:0 methyl ester as the internal standard according to the AOCS method [59].

### 4.2. Preparation of sPAG by Enzymatic Acidolysis Process under CO_2_SC Using RSM

For the phenolic enzymatic acidolysis with gallic acid, a Speed SFE system model 7071 (Applied Separation) supercritical CO_2_ reactor was used, with sAG and gallic acid as substrates, in an extraction vessel of 10 g. The reactions were catalyzed by the specific lipase from *Thermomyces lanuginosus* (10% concerning the substrate) and a 2²-screening factorial design of two factors (CO_2_SC pressure and CO_2_SC temperature, Table 1). The range of the design variables was 40–80 °C (SC temperature) and 78–300 bar (SC pressure). The duration of each static acidolysis reaction was 6 hours, then the final product was extracted for 1 hour and stored at −80 °C until further analysis.

### 4.3. Purification of sPAG by Neutralization with NaOH

The sPAGs obtained from each of the enzymatic acidolysis reactions of the CO_2_SC 2²- screening factorial design were purified by neutralizing with NaOH [60] to remove the remaining fatty acids from the reaction and then collected in hexane for GLC analysis. For this, mixtures were made with ethanol and phenolphthalein, titrations with sodium hydroxide, and washings with hexane. The purification status of each sample was followed by TLC.

### 4.4. Analysis of the Formation of sPAG with Gallic Acid by HPLC-UV

This analysis was performed to corroborate the formation of sPAG with gallic acid, according to the methodology proposed by Sabally et al. [44,47] with modifications. Briefly, the samples obtained from each of the seven experiments (Table 1) were analyzed by high-performance liquid chromatography (HPLC). An Agilent 1100 HPLC equipment (Agilent Technologies Inc., CA-USA) was used, equipped with an ultraviolet detector to detect the acylglycerols (215 nm), as well as gallic acid bound to these structures (280 nm). The acidolysis yield (% synthesis) was calculated as the total peak area of the sPAG at 280 nm, divided by the total area of the gallic acid in the blank multiplied by 100 [44].

### 4.5. Optimization of sPAG Synthesis by RSM

The 2²-screening factorial experimental design based on RSM was used where the effect of two independent variables was studied: SC pressure (78–300 bar) and SC temperature (40–60 °C) on the response variables EPA, DHA, EPA + DHA (g/100 g TFA) and sPAG synthesis (% acidolysis). The design had seven total experimental runs, of which three corresponded to central points that allowed estimating the experimental error (Table 1). The assays were performed in a randomized way to minimize the effect of variability on the observed responses. Process optimization was performed using RSM as described by Myers and Montgomery [61]. The data allowed us to build predictive polynomial models in terms of their regression coefficients for the independent variables and to establish the combination of the variables that allowed obtaining a maximum content of EPA, DHA, EPA + DHA and sPAG synthesis. A mathematical model was obtained from RSM, in which the effect of the independent variables set could be predicted, where Y is the estimated response for the first-order model(Equation 5):

Y = β0 + Σβiχi + Σβiiχj+ ΣΣβijχiχj + ɛ(5)

β0, βi, βii, βij represent the intercept, linear and interaction regression coefficients, respectively; Xi and Xj are the independent variables and ɛ correspond to the random error [61]. The regression coefficients were obtained through multiple regression analysis considering a significance level of *p* < 0.05. An ANOVA of the regression parameters and the fitted model was performed with a significance level of *p* < 0.05. The Statgraphics Centurion XVI-2016 statistical program (Stat Point Technologies, Inc., Rockville, MD, USA) was used. Finally, the sPAG was experimentally validated by applying the proposed theoretically optimum conditions. The optimal sPAG obtained was stored under a nitrogen atmosphere until it was analyzed.

### 4.6. Animals

Male mice (C57BL/6J, 6-week aged, 20.0 ± 2.0 g) were obtained from the Instituto de Salud Pública, Ñuñoa, Santiago, Chile. Mice were kept in a temperature-controlled room with a 12 h:12 h light/dark cycle. Diet (control or high fat diet) was given ad libitum for four weeks. Male C57BL/6 adult mice were fed with a HFD (60% fat, 20% protein, and 20% carbohydrates, research diet #12492) for three months and then were aleatory divided into five groups in which different supplementation was provided: Group A, sunflower oil (200 µL per day); group B, gallic acid (200 µL per day, 70 mg/kg); group C, DRCSO (200 µL per day, DHA 150 mg/kg); group D, sAG (200 µL per day) and group E, sPAG (200 µL per day). The supplements were administered orally using a syringe, to avoid using a catheter, considering that the treatment was administered daily and for a month. Animals were maintained following the NIH Guide for the Care and Use of Laboratory Animals, and protocols were approved by the bioethical committee of the Faculty of Medicine, Santiago, University of Chile (Bioethical protocol number CBA1001, FMUCH).

The doses of gallic acid supplied (70 mg/kg) were defined according to the same previous test and related literature [28,29,51], taking into account that the maximum dose may be 100 mg/kg weight.

### 4.7. Biological Samples

At the end of the experimental period, mice were euthanized after four hours of fasting. Serum and tissues were collected immediately after euthanasia. Serum was obtained after centrifugation of the blood (2000× *g* at 4 °C for 15 min). Hepatic tissue was dissected, weighted, and then fixed in paraffin for histological studies.

### 4.8. Measurements of Serum Parameters

Levels of serum biochemical markers were measured by dry chemistry technology (SPOTCHEM™ EZ, Minneapolis, MN, USA). Serum insulin concentrations were determined by a commercially available immunoassay specific for mice (Mercodia, Uppsala, Sweden).

### 4.9. Glucose Tolerance Test

An intraperitoneal glucose tolerance test (iGTT) was performed using 20% glucose solution, adjusting to 2 g/kg. Then, blood samples were collected from the tail vein using a capillary tube at 15, 30, 60, 90, and 120 min after intraperitoneal injection of glucose.

### 4.10. Statistics

A statistical analytical system was used for multiple regression analysis, analysis of variance (ANOVA), canonical analysis, and ridge maximum data analysis in the response surface regression (RSREG) procedure. Estimated response surfaces and contours of estimated response surfaces were developed using the fitted quadratic polynomial equations obtained from RSREG analysis and holding the process variables with the minor effect on the response at a constant value and changing the levels of the other two variables. Analyses were performed in triplicate. The 95% confidence intervals of each quality parameter were calculated, considering the number of replicates and the standard deviation of each sample. The lack-of-fit test was performed by comparing the variability of the current model residuals with the variability between observations at replicate settings of the factors. Statgraphics^®^Centuriun XVI-2011 software (StatPoint Technologies, Inc., Rockville, MD, USA) was used.

## 5. Conclusions

We conclude that both gallic acid and a sPAG rich in EPA and DHA could be a promissory strategy in the structural lipid field to improve the metabolic alterations induced by obesity. However, for technical performance and development, a dose adjustment and long-time monitoring of chronic effects would be crucial.

## Figures and Tables

**Table 1:** 2²-screening factorial design (RSM) of two factors and seven experimental runs to optimize the synthesis of sPAG (% acidolysis), EPA, DHA, EPA + DHA content (g/100 g TFA) in the enzymatic acidolysis process.

	Independent Variables		Response Variables			
Run	Supercritical Temperature (°C)	Supercritical Pressure (Bar)	EPA (g/100 g TFA)	DHA (g/100 g TFA)	EPA + DHA (g/100 g TFA)	Synthesis of sPAG (% Acidolysis)
1	40	78	10.745	7.504	18.249	28.1
2	80	78	11.089	8.421	19.510	33.3
3	40	300	10.730	7.837	18.567	21.4
4	80	300	11.779	8.636	20.415	23.9
5	60	189	11.043	8.337	19.381	24.2
6	60	189	11.016	8.223	19.239	23.8
7	60	189	11.088	8.009	19.097	24.4

**Table 2:** Validation of FA composition and quantification of sPAG by GLC (g/100 g TFA).

Systematic Name	Abbreviated Name	sAG	sPAG
Lauric acid	C12:0	N/I	N/I
Myristic acid	C14:0	2.15 ± 0.00	1.66 ± 0.00
Palmitic acid	C16:0	10.60 ± 0.00	7.90 ± 0.01
Palmitoelaidic acid	C16:1 9t	0.21 ± 0.00	0.22 ± 0.00
Palmitoleic acid	C16:1 9c	3.39 ± 0.00	3.21 ± 0.00
Heptadecanoic acid	C17:0	N/I	N/I
Heptadecenoic acid	C 17:1 10c	0.44 ± 0.00	0.57 ± 0.00
Stearic acid	C18:0	3.17 ± 0.00	2.54 ± 0.00
Trans-vaccenic acid	C 18:1 11t	0.47 ± 0.01	0.61 ± 0.00
Oleic acid	C18:1 9c	34.66 ± 0.03	27.60 ± 0.03
Cis-Vaccenic acid	C18:1 7c	2.74 ± 0.00	2.22 ± 0.00
Linoleaidic acid	C18:2 9t 12t	0.75 ± 0.01	0.94 ± 0.01
Linoleic acid	C18:2 9c 12c	15.48 ± 0.00	18.12 ± 0.00
Gamma linolenic acid	C18:3 6c 9c 12c	0.37 ± 0.00	0.44 ± 0.00
5-Eicosanoic acid	C 20:1 5c	N/I	0.44 ± 0.00
8-Eicosanoic acid	C 20:1 8c	N/I	N/I
11-Eicosanoic acid	C 20:1 11c	1.94 ± 0.00	1.57 ± 0.00
α-Linolenic acid	C 18:3 9c 12c 15c	4.78 ± 0.00	5.95 ± 0.01
Eicosadienoic acid	C 20:2 11c 14c	0.90 ± 0.00	0.78 ± 0.00
Behenoic acid	C 22:0	N/I	N/I
Eicosatrienoic acid	C 20:3 11c 14c 17c	1.29 ± 0.00	1.08 ± 0.01
Erucic acid	C 22:1 13c	N/I	N/I
Arachidonic acid	C 20:4 5c 8c 11c 14c	N/I	N/I
Docosadienoic acid	C 22:2 13c 16c	1.07 ± 0.00	1.41 ± 0.00
Eicosapentaenoic acid	C 20:5 5c 8c 11c 14c 17c	5.92 ± 0.01	11.11 ± 0.03
Nervonic acid	C 24:1 9c	N/I	N/I
Docosatetraenoic acid	C 22:4 7c 10c 13c 16c	0.49 ± 0.00	0.67 ± 0.00
Docosapentaenoic acid	C 22:5 7c, 10c 13c 16c 19c	2.00 ± 0.00	2.52 ± 0.00
Docosahexaenoic acid	C 22:6 4c 7c 10c 13c 16c 19c	7.18 ± 0.02	8.44 ± 0.00
Total saturated fatty acids (TSFA)		15.92	12.10
Total monounsaturated fatty acids (TMUFA)		43.85	36.44
Total polyunsaturated fatty acids (TPUFA)		40.23	51.46
Total n-3 long-chain PUFA (n-3 TLCPUFA)		16.39	23.15
Total n-3 fatty acids (n-3 TFA)		21.17	29.10
EPA + DHA		13.10	19.55

## Data Availability

Not applicable.

## Supplementary Materials

The following supporting information can be downloaded at: https://www.mdpi.com/article/10.3390/molecules27227702/s1, Figure S1: Thin-layer chromatography separation: (A) free fatty acids standard, (B) DRCSO, (C–F) sPAG non purified, (G) sPAG purified and (H) gallic acid standard.; Table S1: Optimal values of the independent variables SC pressure and SC temperature to maximize the responses EPA, DHA and EPA+DHA content (g/100 g TFA), sPAG synthesis (%) and the joint optimization of all the variables; Refs. [37,44,45,62] is cited in the Supplementary Materials.

## Sample Availability

Samples of the compounds are not available from the authors.
