# Supplementary material for: Effect of Structured Phenolic Lipids with EPA/DHA and Gallic Acid against Metabolic-Associated Fatty Liver Disease (MAFLD) in Mice
Source: Molecules. 2022 Nov 9;27(22):7702. doi: 10.3390/molecules27227702 (PMC9696657; doi:10.3390/molecules27227702)
Supplement: Supplementary file 1 [file molecules-27-07702-s001.zip › molecules-1973603-supplementary.pdf]

Supplementary Materials

Optimization of the acidolysis process for obtaining sPAG by RSM

Table S1 shows the process variables optimization for obtaining sPAG with EPA, DHA, and EPA+DHA (g/100 g TFA) and phenolic acidolysis (%).

Table S1: Optimal values of the independent variables SC pressure and SC temperature to maximize the responses EPA, DHA and EPA+DHA content (g/100 g TFA), sPAG synthesis (%) and the joint optimization of all the variables.

Optimization	Supercritical temperature (°C)	Supercritical pressure (bar)	Stationary point	Optimal predicted value
**EPA**	80	300	Maximum	11.76
**DHA**	80	300		8.67
**EPA+DHA**	80	300		20.44
**sPAG synthesis**	80	78		32.24
**Joint optimization**	80.0	78.0	Maximum	**EPA:** 11.15<br>**DHA:** 8.38<br>**EPA+DHA:** 19.53<br>**sPAG synthesis:** 32.24

Identification of DRCSO, *n-3 PUFAC and sPAG by TLC.*

All the samples were identified by TLC on Silica gel 60 F254 plates (Merck). A solution of chloroform, acetone, and glacial acetic acid (96/4/1, v/v/v, respectively) was used as eluent. [1] The elution order on the chromatographic plate from bottom to top was produced according to their decreasing polarity: MG, DG, and TG [2]. In order to visualize the lipid structures, the plates were stained with iodine solution.

Thin-Layer Chromatography (TLC) of optimal validated sPAG

TLC separation of purified and non-purified sPAG is shown in Figure S1. The presence of products resulting from enzymatic acidolysis reactions with gallic acid under CO_2SC conditions (i.e., monoglycerols (MG), diacylglycerols (DG), triacylglycerols (TG), phenolic monoglycerols and phenolic diacylglycerols were observed according to the polarity degree [3]. As expected, MG molecules migrated the least, while TG molecules migrated the fastest, according to previous research [4]. The phenolic compounds, corresponding to sPAG monoglycerols and sPAG diacylglycerols migrated slower than their corresponding triacylglycerols [1] and the gallic acid standard did not elute probably due to its high polarity. TLC analysis confirmed the presence of sPAG in bands C-F (non-purified) and G (purified), also showing that purification with NaOH removes a part of MG and phenolic MG. The elution order observed in DRCSO showed that FA are predominantly found in the form of TG in DRCSO, characteristic of refined oils.

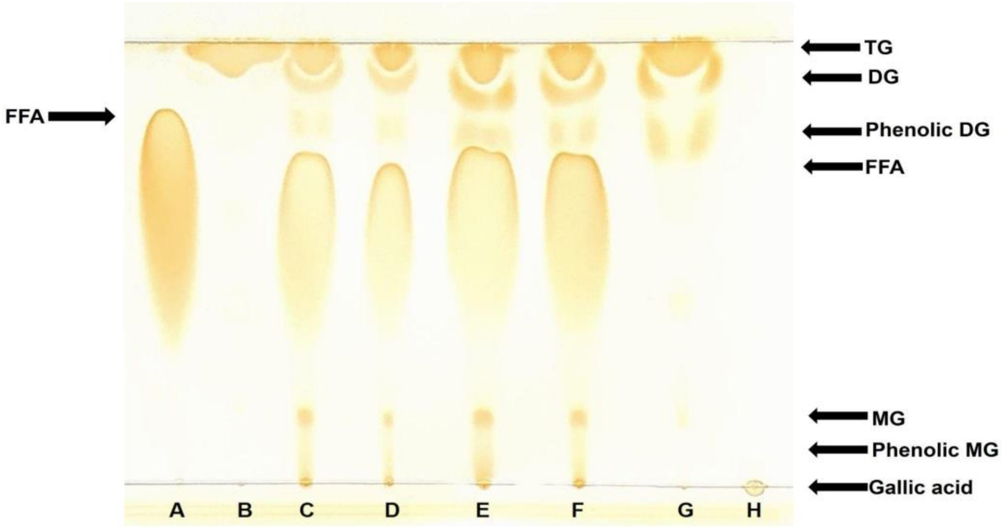

Figure S1: Thin-layer chromatography separation: (**A**) free fatty acids standard, (**B**) DRCSO, (**C–F**) sPAG non purified, (**G**) sPAG purified and (**H**) gallic acid standard.

References:

[1]Sabally, K.; Karboune, S.; St-Louis, R.; Kermasha, S. Lipase-catalyzed transesterification of dihydrocaffeic acid with flaxseed oil for the synthesis of phenolic lipids. *J. Biotechnol.* **2006**, *127*, 167–176. https://doi.org/10.1016/j.jbiotec.2006.06.010.

[2]Wanasundara, U. N. and Shahidi, F. Positional distribution of fatty acids in triacylglycerols of seal blubber oil. *J. food lipids.* **1997**,*4*, 51–64. https://doi:10.1111/j.1745-4522.1997.tb00080.x.

[3]Dovale-Rosabal, G.; Rodríguez, A.; Espinosa, A.; Barriga, A.; Aubourg, S.P. Synthesis of EPA- and DHA-enriched structured acylglycerols at the *sn*-2 position starting from commercial salmon oil by enzymatic lipase catalysis under supercritical conditions. *Molecules* **2021**, *26*, 3094. https://doi.org/10.3390/molecules26113094.

[4]Haq, M.; Park, S. K.; Kim, M. J.; Cho, Y. J. and Chun, B. S. Modifications of Atlantic salmon by-product oil for obtaining different ω-3 polyunsaturated fatty acids concentrates: An approach to comparative analysis. *J. Food Drug Anal.* **2018**, *26*, 545-556. https://doi:10.1016/j.jfda.2017.05.006.
